# Humanism Rounds: A Multifaceted “Back to Bedside” Initiative to Improve Meaning at Work for Internal Medicine Residents

**DOI:** 10.1007/s40670-024-02017-9

**Published:** 2024-03-13

**Authors:** Jennifer M. Kaplan, Suchi Agrawal, Disha Kumar, Ann Xu, Kristen A. Staggers, Anna G. Symmes, Reina U. Styskel, Brett Styskel, Anoop Agrawal, Stacey R. Rose

**Affiliations:** 1 Section of Endocrinology, Department of Internal Medicine, Baylor College of Medicine, Houston, TX USA; 2 Department of Internal Medicine, Baylor College of Medicine, Houston, TX USA; 3 Section of Gastroenterology, Department of Internal Medicine, Baylor College of Medicine, Houston, TX USA; 4 Institute for Clinical and Translational Research, Baylor College of Medicine, Houston, TX USA; 5 Department of General Internal Medicine, East Carolina University, Greenville, NC USA; 6 Division of Hospital Medicine, Cleveland Clinic Foundation, Cleveland, USA; 7 Department of Gastroenterology, Hepatology and Nutrition, Cleveland Clinic Foundation, Cleveland, OH USA; 8 Departments of Medicine and Pediatrics, Baylor College of Medicine, Houston, TX USA; 9 Section of Infectious Diseases, Department of Internal Medicine, Baylor College of Medicine, Houston, TX USA; 10 Center for Professionalism, Baylor College of Medicine, Houston, TX USA

**Keywords:** Burnout, Humanism in medicine, Learning environment, Resident wellness

## Abstract

**Introduction:**

Burnout is an increasingly prevalent problem among resident physicians. To address this problem, the Accreditation Council on Graduate Medical Education (ACGME) created the Back to Bedside initiative, supporting resident-driven projects focused on increasing direct interactions with patients. In 2017, Baylor College of Medicine (BCM) Internal Medicine Residency received a Back to Bedside grant to develop and implement “Humanism Rounds,” a multifaceted program which sought to promote personal connections between residents and patients and foster reflection about patients’ non-clinical stories, with the hopes of reducing burnout and increasing residents’ sense of meaning at work.

**Materials and Methods:**

Between 2018 and 2020, internal medicine residents were instructed on and encouraged to participate in Humanism Rounds. The program included three components: taking a “human history,” bedside rounds focused on non-clinical concerns, and sharing patient stories with colleagues (“celebrations”). Residents were surveyed using institutional and ACGME surveys regarding burnout, meaning at work, and the clinical learning environment.

**Results:**

Three hundred eleven institutional (response rate, 74%) and 328 AGCME (response rate, 78%) surveys were completed and analyzed. Residents who actively engaged with Humanism Rounds reported more meaning and fulfillment at work (*p* < 0.001). During the period of this project, ratings of the learning environment and personal callousness improved among subgroups of residents.

**Conclusions:**

Baylor College of Medicine Internal Medicine residents who engaged with Humanism Rounds reported more meaning and fulfillment in their work. This program describes a low-cost model for other specialties and institutions to strengthen human connections and improve residents’ experience during training.

**Supplementary Information:**

The online version contains supplementary material available at 10.1007/s40670-024-02017-9.

## Introduction 

Burnout affects medical residents nationwide, leading to poor resident wellbeing, career dissatisfaction, and decreased quality of patient care [1, 2]. The rates of burnout among residents range from 27 to 75%, with high rates noted in obstetrics and gynecology (75%), internal medicine (63%), and general surgery (40%) with the lowest rate among family medicine residents (27%) [3]. Research into burnout during residency has focused on a variety of contributing factors including sleep deprivation, time away from work, social relatedness, and resilience [4–7]. Perception of meaning in work is thought to mitigate burnout by reducing depersonalization and emotional exhaustion and increasing a sense of personal achievement [2, 7]; clinicians who feel more connected to their patients report an enhanced sense of meaning in their work [8]. Additionally, interventions focused on storytelling, narrative medicine, or reflective practice may also promote wellbeing among trainees and healthcare workers [9–11].

To address resident burnout, the Accreditation Council on Graduate Medical Education (ACGME) developed the Back to Bedside initiative: a competitive grant program soliciting resident-driven projects focused on encouraging more direct resident–patient interaction [12, 13]. The Baylor College of Medicine (BCM) Internal Medicine Residency was awarded one of the original Back to Bedside grants from the ACGME to support a novel initiative: Humanism Rounds.

Humanism Rounds utilized a three-pronged approach of “human history” taking, bedside rounds, and sharing patient stories with colleagues (“celebrations”) to allow trainees to enhance connections with patients and colleagues. More specifically, the Humanism Rounds intervention aimed to foster humanistic connections with patients and to promote reflection and storytelling with peers to increase meaning in work and reduce burnout among internal medicine residents at BCM.

## Materials and Methods

Humanism Rounds was accepted into the ACGME Back to the Bedside collaborative in October 2017. The project was conducted within the BCM Internal Medicine Residency, a large academic multi-center training program in Houston, Texas, with approval from the BCM Institutional Review Board.

### Project Components

**Fig. 1:**
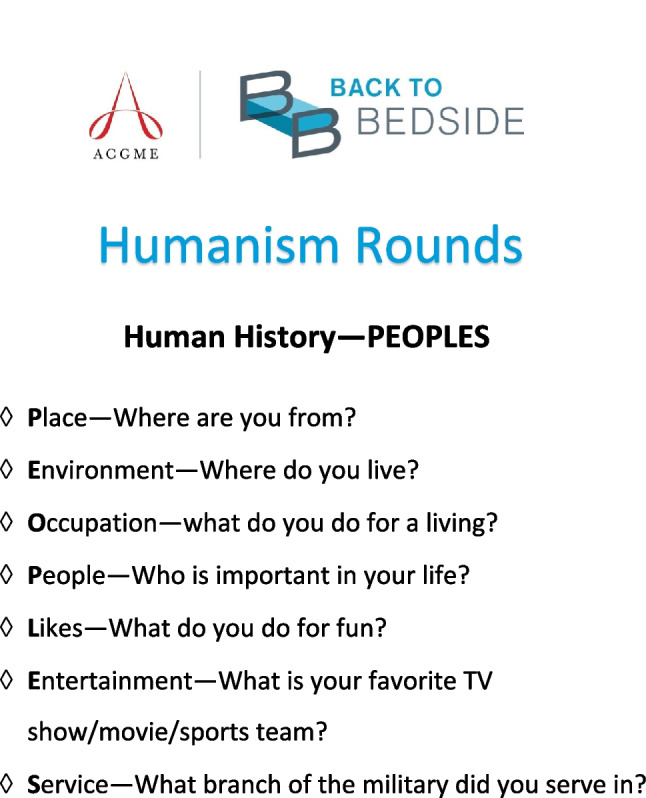
Taking a human history

The project consisted of three components: “Human Histories,” “Humanism Rounds,” and “Celebrations.” “Human Histories” focused on eliciting aspects of a patient’s history that are not directly related to his or her medical condition, such as hobbies and personal interests (see Fig. 1). Human Histories were conducted during Humanism Rounds in which a resident would select one patient to visit at the bedside for 10–20 min as a team or one-on-one. Patient care coverage was provided by attending physicians and chief medical residents to enable residents to have uninterrupted conversations during Humanism Rounds. Celebrations were monthly, hospital-wide gatherings when all residents were invited to share stories from their Humanism Rounds experiences during regularly scheduled didactic sessions.

### Project Phases

#### Pilot Phase

**Fig. 2:**
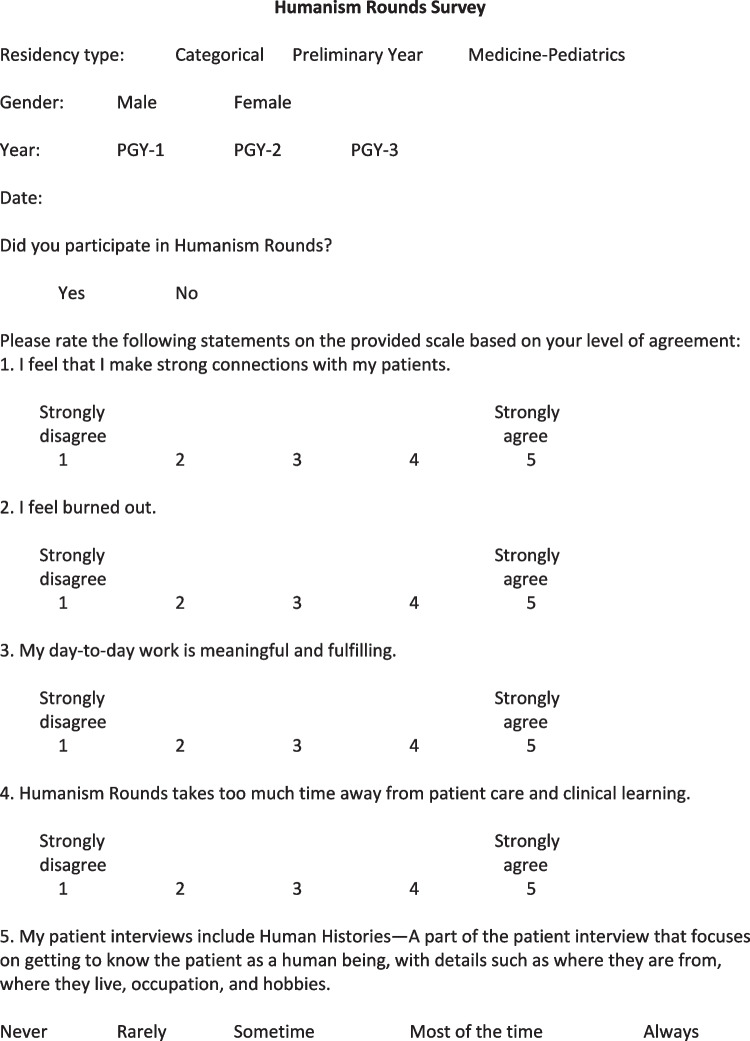
Humanism Rounds Survey (institutionally created)

Between January and March 2018, a pilot phase of the initiative was conducted to ensure that resident participation in Humanism Rounds did not detract from patient care or create excess resident burden. Thirty volunteers were recruited via email and surveyed using an institutional questionnaire which solicited feedback on burnout, meaning at work, and feasibility of integrating the intervention components into daily practice (see Fig. 2).

#### Intervention Phase

Following the pilot phase, the intervention was expanded to include all residents on inpatient rotations at any affiliated teaching hospital (including Ben Taub General Hospital, the Michael E. Debakey Veterans Affairs Medical Center, Baylor St. Luke’s Medical Center, or MD Anderson Cancer Center). All residents were introduced to the Humanism Rounds project components either during regular didactic sessions (upper level residents) or during orientation (interns). An instructional PowerPoint explained the background for the project and taught residents how to elicit a Human History using the PEOPLES mnemonic: P, place; E, environment; O, occupation; P, people; L, likes; E, entertainment; S, service (military, for veteran patients) (see Appendix A). Interns and residents were also provided with a pocket card with guidance for taking a Human History (see Fig. 1). Though participation was optional, residents were regularly encouraged to incorporate Human Histories and to participate in Humanism Rounds individually or with their teams. Celebrations were held at the end of each month, facilitated by chief residents or Back to Bedside resident ambassadors. Suggested discussion prompts were provided; the prompts encouraged residents to consider their experiences with a range of emotions, thus facilitating self-reflection [11] (see Table 1).

**Table 1:** Suggested discussion prompts during Humanism Rounds celebrations

Tell a story about:
• Your Humanism Rounds experience
• A patient who made you feel hopeful
• A patient who made you laugh
• A patient who made you sad
• A patient you learned from
• A patient who died

### Data Collection

Data were collected using institutionally created Humanism Rounds surveys (see Fig. 2) as well as a compilation “Back to Bedside Assessment Tool” curated by ACGME for use by all Back to Bedside initiatives. The institutionally created survey asked specific questions regarding the Humanism Rounds intervention, such as the extent to which a resident was incorporating Human Histories into their patient interviews, and whether the Humanism Rounds initiative impeded patient care or clinician learning. The survey also asked residents to rate their level of burnout and meaning in work. The Back to Bedside Assessment Tool provided by ACGME included validated assessments of burnout, meaning at work, and the learning environment, such as the Maslach Burnout Inventory [14], the Work and Meaning Inventory [15], the Subjective Vitality Scale [16], and the Learning Climate Questionnaire [17]. Due to copyright constraints, the compilation Back to Bedside Assessment Tool could not be included as part of this report.

To assess the effectiveness of the intervention, residents were asked to complete baseline surveys (both institutionally created and the ACGME Back to Bedside Assessment Tool) in July to August 2018, with follow-up surveys conducted in January and February of 2019 and 2020. Although residents were also surveyed in 2020, response rates were limited due to COVID-19-related interruptions in residency activities, and the data was thus omitted from analysis. Resident participation in the Humanism Rounds program, including survey completion, was voluntary.

### Statistical Analysis

Resident characteristics, survey responses, and survey scores were summarized by median with minimum and maximum values or by frequency with percentage. The Wilcoxon rank-sum, Fisher’s exact, or Chi-square test was used to make comparisons as appropriate. Since resident identification was unknown, we could not be sure the same residents completed surveys both pre- and post-interventions, so any analyses which compared between the two survey time points were done within each level of training for the assumption of independent observations to be satisfied. *p* < 0.05 was used for statistical significance.

## Results

Among 418 internal medicine residents in the BCM Internal Medicine Residency during the intervention timeframe, 311 (response rate, 74%) completed institutionally created surveys, and 328 (response rate, 78%) completed ACGME surveys in 2018 or 2019. Most respondents were categorical residents, with a higher proportion of interns (post-graduate year 1 or PGY-1s) as compared to PGY-2s or PGY-3s (see Table 2).

**Table 2:** Resident demographics (*N* = 327)^a^

Residency type (*N* = 324)	*n* (%)
Categorical	284 (87.7)
Preliminary	29 (9.0)
Medicine-pediatrics	11 (3.4)
Gender (*N* = 326)	
Male	193 (59.2)
Female	133 (40.8)
Year of training (*N* = 327)	
PGY-1	152 (46.5)
PGY-2	95 (29.1)
PGY-3	78 (23.9)
PGY-4	2 (0.6)

^a^Per respondents on ACGME Back to Bedside Assessment Tool, 2018–2029; 328 residents responded to the survey, but one response did not denote any demographic information and thus was excluded from this table

### Institutionally Created Surveys (See Table 3)

**Table 3:** Meaning and fulfillment at work among residents who did versus did not participate in Humanism Rounds^a^

	Participated (*N* = 109)	Did not participate (*N* = 43)	*p*-value
Residents who agreed Humanism Rounds improved their sense of meaning and fulfillment at work, *n* (%)	54 (49.5%)	4 (9.3%)	< 0.001

^a^Results from institutionally created survey data from 2019

^b^*p*-value calculated with Fisher’s exact test

After the intervention was introduced, the percentage of residents who felt the Back to Bedside initiative had improved their sense of meaning, and fulfillment at work was higher among those who self-reported participation in Humanism Rounds compared to those who did not participate (49.5% vs 9.3%, *p* < 0.001). This finding remained statistically significant when evaluating within PGY-1 (*p* < 0.001) and PGY-2 (*p* = 0.020) residents. Although not statistically significant, more PGY-3 residents reported improved meaning and fulfillment at work if they participated in Humanism Rounds compared to those who did not participate (50% vs 20%, *p* = 0.101).

### ACGME Back to Bedside Assessment Tool (See Table 4)

**Table 4:** Select ACGME Back to Bedside Assessment Tool responses before and after Humanism Rounds

	**Spring 2018 (before)**		**Spring 2019 (after)**		
**Survey**	**Group (** ***N*** **)**	**Median [Min, Max]**	**Group (** ***N*** **)**	**Median [Min, Max]**	***p*** **-value** ^**a**^
Learning environment score	PGY-1 (93)	5.3 [1.8, 7.0]	PGY-1 (59)	5.8 [3.2, 7.0]	**0.001** ^**b**^
	PGY-2 (38)	5.3 [2.8, 7.0]	PGY-2 (57)	5.5 [2.2, 7.0]	0.637
	PGY-3 (28)	5.6 [4.2, 7.0]	PGY-3 (50)	5.7 [2.2, 7.0]	> 0.99

	**Group (** ***N*** **)**	**Yes** ***N*** ** (%)**	**Group (** ***N*** **)**	**Yes** ***N*** ** (%)**	
I have become more callous towards people once a week or more since I took this job	PGY-1 (93)	11 (11.8)	PGY-1 (59)	12 (20.3)	0.169
	PGY-2 (38)	7 (18.4)	PGY-2 (57)	19 (33.3)	0.159
	PGY-3 (28)	10 (35.7)	PGY-3 (50)	7 (14.0)	**0.043** ^**b**^
I feel burned out once a week or more from my work	PGY-1 (93)	20 (21.5)	PGY-1 (59)	16 (27.1)	0.440
	PGY-2 (38)	11 (28.9)	PGY-2 (57)	23 (40.4)	0.283
	PGY-3 (28)	10 (35.7)	PGY-3 (50)	14 (28.0)	0.610

^a^Median comparisons estimated using the Wilcoxon rank-sum test and categorical comparisons estimated using Fisher’s exact or Chi-square test

^b^Comparison meets threshold for statistical significance

Among PGY-1 residents, the learning environment score as assessed on the Learning Climate Questionnaire [16] was higher in 2019 than 2018 (median 5.8 vs 5.3, *p* = 0.001). This score is derived from a composite of questions regarding the perceived relationship between the resident and their immediate supervisor, with higher scores indicating a supportive relationship and lower scores indicating a negative relationship. For PGY-3 residents, the percentage of those who became more callous towards patients (part of the Maslach Burnout Inventory [13]) was lower in 2019 compared to 2018 (14.0% vs 35.7%, *p* = 0.043). No other scores on the ACGME Back to Bedside Assessment Tool differed significantly between time points within PGY-1, PGY-2, or PGY-3 residents. Specifically, the rate of burnout per the modified Maslach Burnout Inventory [14] did not differ between time points during the study.

## Discussion

Across all years of training, residents who actively engaged in Humanism Rounds reported that the practice improved their sense of meaning and fulfillment at work. Due to the voluntary nature of this intervention and competing clinical and personal commitments, not all residents participated actively. Nonetheless, all residents were introduced to the components of the Humanism Rounds program (taking a Human History; Humanism Rounds; and monthly Celebrations). Institutional support of Humanism Rounds was evidenced by integrating the project into residency orientations and hosting Celebrations during regularly scheduled didactic sessions. This awareness of Humanism Rounds and the support of the residency program leadership may have conferred benefit to those residents who did not actively engage in Humanism Rounds. This may explain the smaller, yet notable, reported improvement in meaning and fulfillment at work among this group.

Sir William Osler remarked that “the good physician treats the disease; the great physician treats the patient who has the disease.” This guidance, however, is challenging to enact in a time when internal medicine residents spend more time completing documentation than they spend directly with patients [18]. Our findings support efforts to improve meaning in the clinical learning environment by encouraging and allowing residents to appreciate their patients’ multifaceted lives. A PGY-3 participating in the Humanism Rounds program expressed this sentiment with the following comment: “Learning about who my patients are, what they’ve achieved, what struggles they’ve been through outside of their illness does more than add to the overall social history. It completes the picture of who you’re taking care of and why treating them means something on a truly holistic level.” Residents did not feel that this practice detracted from patient care duties based on feedback from residents during the pilot phase. While the ability to increase time spent with patients is limited, Humanism Rounds enables residents to spend more quality time with patients.

After establishing the Humanism Rounds program, PGY-3s reported they were less callous towards others and PGY-1s rated the learning environment of the residency program as improved. While many factors compose the clinical learning environment, several aspects of the intervention established Humanism Rounds as part of the environment for the BCM Internal Medicine Residency. By consistently hosting Celebrations during didactic conferences, residents were given protected time to engage in Humanism Rounds, and the residency program leadership indicated its support of the practice. These findings suggest a multilayered effect: the individual finds more meaning and fulfillment at work by connecting with patients, and the culture of the residency program is affected by the consistent implementation and endorsement from program leadership of Humanism Rounds.

Although previous studies have shown that an increased sense of meaning at work and a positive work environment are associated with decreased burnout [1], the number of residents who described themselves as burned out did not change after introducing Humanism Rounds. This finding should not be interpreted to indicate that the program failed to impact burnout; rather, we must consider that although Humanism Rounds facilitates a personal connection with patients, the program did not address other factors, such as workload, duty hours, and administrative tasks known to contribute to burnout in residency. We suspect that larger investments in restructuring the clinical learning environment would be needed to impact overall burnout ratings.

Beyond the quantitative results derived from both the institutionally created survey and ACGME Back to Bedside Assessment Tool, the logistical implementation of this project brought to light several important lessons:

1. Residents found that eliciting a human history independently rather than as a team was easier to integrate into their daily workflow. This also fostered a more comfortable atmosphere in the patient’s room.
2. Hospital-wide Celebrations facilitated sustainability of the project and have become part of the fabric of the residency program. These sessions allowed for personal reflection andcreated an opportunity for residents to process experiences as a group. Conducting these discussions during a regularly scheduled didactic session reserved time to engage in this project rather than placing the responsibility on the resident to create time in their personal schedule. It also imparted the message that a focus on humanism in medicine is of equal importance to other educational topics and demonstrated the support of the program and faculty.
3. The continuation of this project was dependent on a clear plan for transferring leadership responsibilities from senior to junior residents. Beyond quantitative measurements, the continuation of Humanism Rounds, which endures at BCM as a resident-driven initiative, is evidence of its success.

In addition to the strengths of this project, there were also limitations. Participation in the intervention and survey completion was voluntary, which may have introduced the potential for responder bias and may limit the ability to apply the study results to all residents in the program. Our statistical analyses were limited by not including resident identifiers, thus requiring us to make comparisons within resident years to keep survey responses independent. Additionally, while the ACGME Back to Bedside Assessment Tool included several validated survey instruments, the institutionally created survey was not formally validated. Finally, the intervention was conducted at a single institution; although the BCM Internal Medicine Residency is one of the largest in the country and included Humanism Rounds and celebrations at multiple hospital venues, the program merits additional study across a larger number of residency program types to comprehensively evaluate its effectiveness.

## Conclusion

Humanism Rounds — the practice of taking a Human History, bedside rounds focused on connecting with patients, and Celebrations for group sharing of patient stories — was launched at the BCM Internal Medicine Residency in 2018 as part of the ACGME Back to Bedside initiative with the goal of fostering stronger connections between resident physicians and their patients. Residents who engaged with Humanism Rounds reported more meaning and fulfillment at work. During the period of this project, ratings of the learning environment and personal callousness improved significantly among certain groups. The rates of burnout did not improve over the course of the study, which was not surprising since the study did not address systems issues such as workload or administrative burdens.

Moving forward, we envision multiple future avenues for the initiative to amplify its effect, such as by including medical students, faculty members, and/or non-physician members of the health care team in both Humanism Rounds and the monthly celebrations. Conducting the monthly Celebrations virtually may also permit expansion of its reach.

Overall, the Back to Bedside Humanism Rounds project provides a model for a low-cost, resident-driven intervention which can enhance resident meaning and fulfillment in the clinical learning environment.

### Supplementary Information

Below is the link to the electronic supplementary material.

Supplementary file1 (PPTX 51.3 KB)

## Data Availability

De-identified summary data that support the findings of this study are available from the corresponding author, [SR], upon reasonable request.
