# Supplementary material for: Humanism Rounds: A Multifaceted “Back to Bedside” Initiative to Improve Meaning at Work for Internal Medicine Residents
Source: Med Sci Educ. 2024 Mar 13;34(3):601–7. doi: 10.1007/s40670-024-02017-9 (PMC11180076; doi:10.1007/s40670-024-02017-9)
Supplement: Supplementary file 1 — Supplementary file1 (PPTX 51.3 KB) [file 40670_2024_2017_MOESM1_ESM.pptx]

## Slide 1
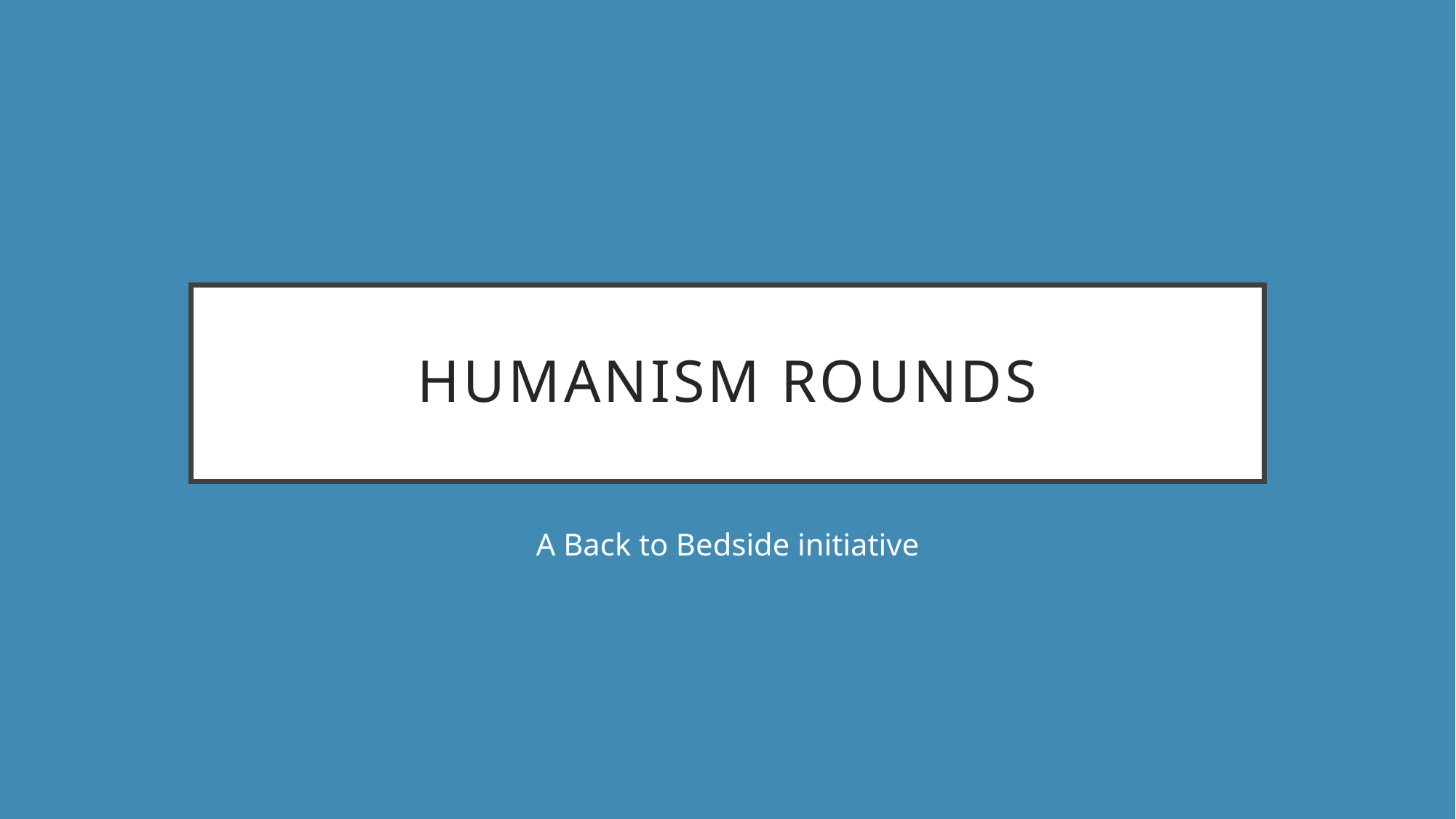

# Humanism Rounds
A Back to Bedside initiative

## Slide 2
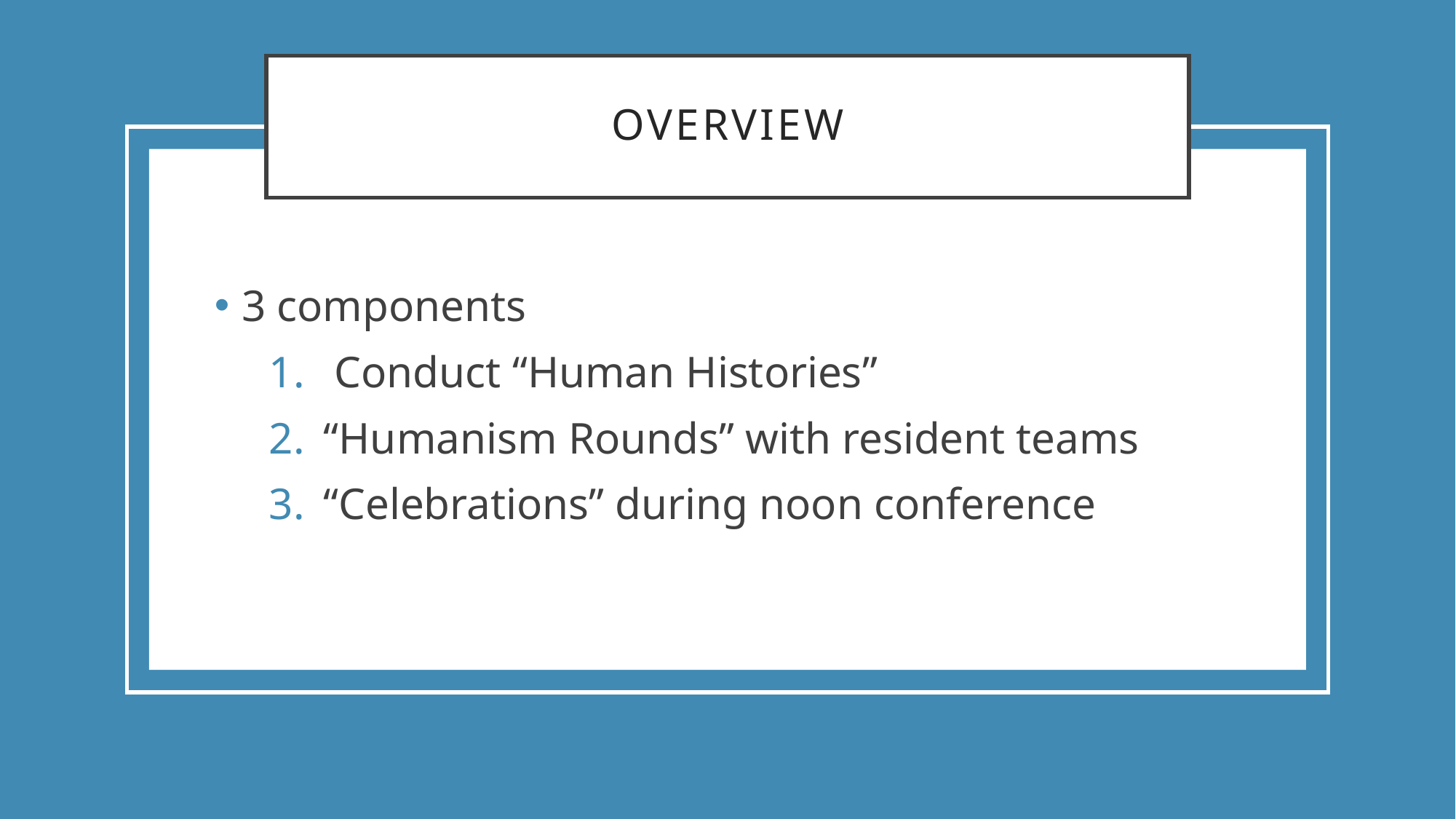

# Overview
3 components
 Conduct “Human Histories”
“Humanism Rounds” with resident teams
“Celebrations” during noon conference

## Slide 3
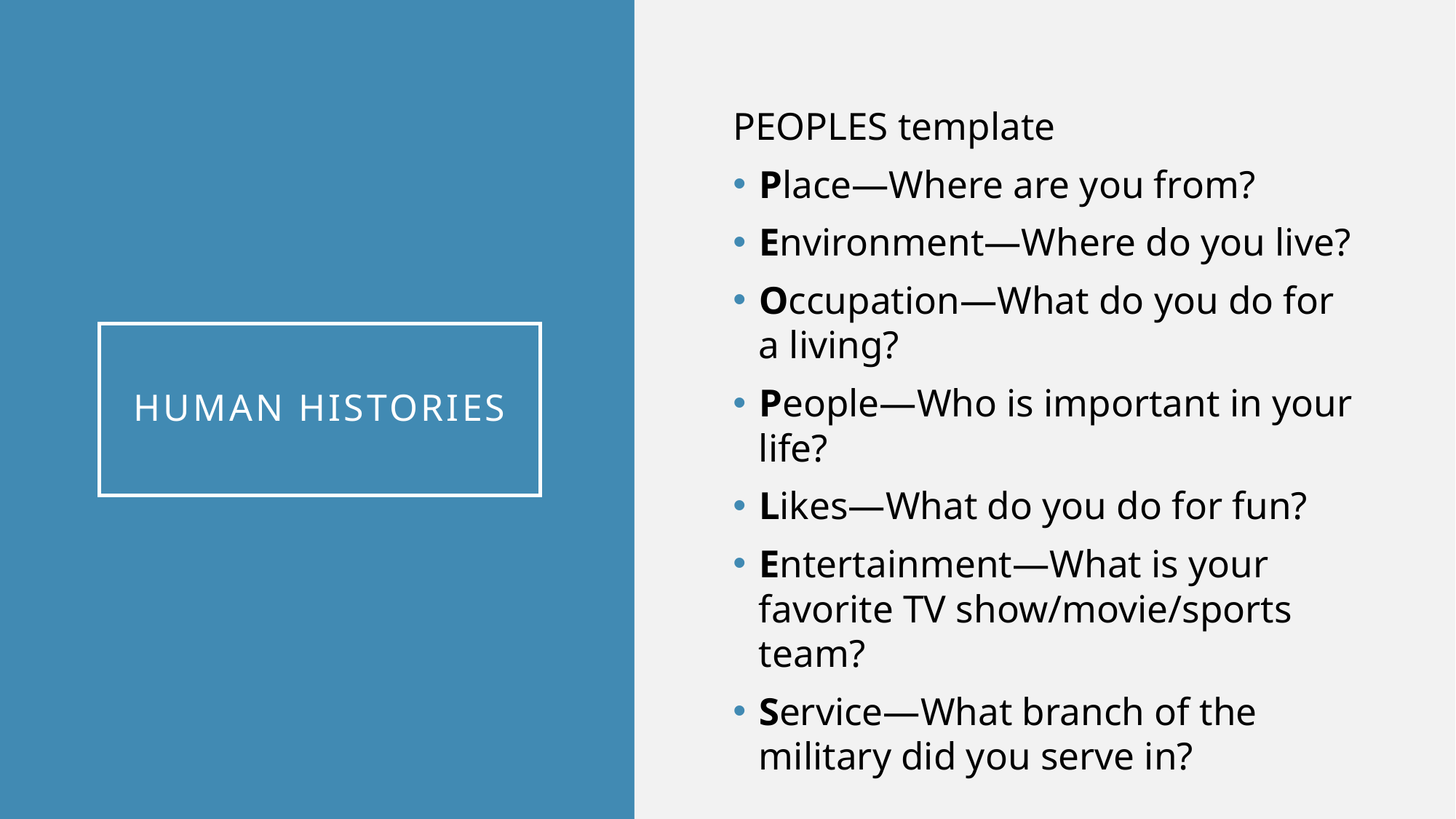

PEOPLES template
Place—Where are you from?
Environment—Where do you live?
Occupation—What do you do for a living?
People—Who is important in your life?
Likes—What do you do for fun?
Entertainment—What is your favorite TV show/movie/sports team?
Service—What branch of the military did you serve in?
# Human Histories

## Slide 4
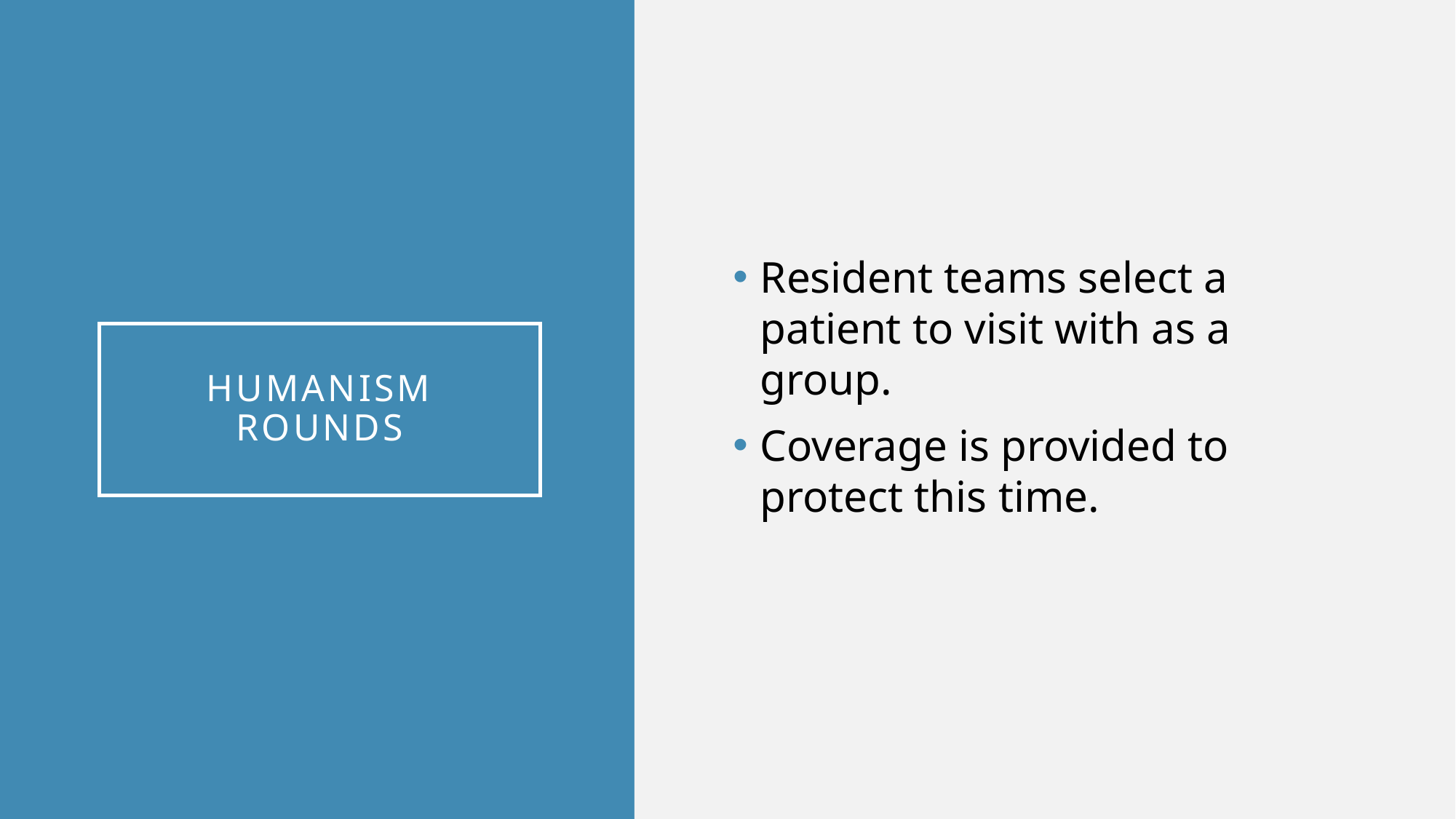

Resident teams select a patient to visit with as a group.
Coverage is provided to protect this time.
# Humanism Rounds

## Slide 5
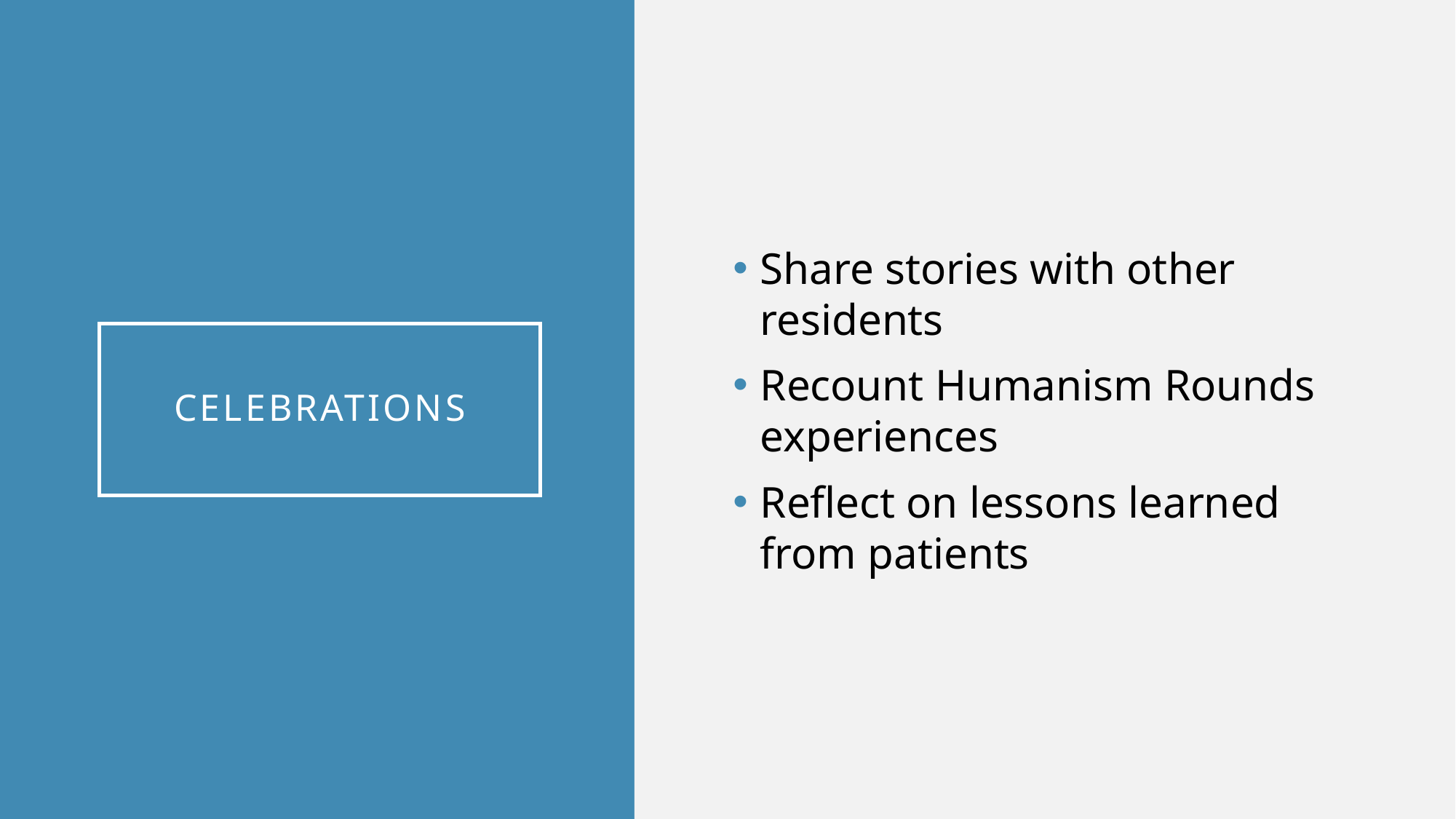

Share stories with other residents
Recount Humanism Rounds experiences
Reflect on lessons learned from patients
# Celebrations
